# Primary osteosarcoma of the breast with abundant chondroid matrix and fibroblasts has a good prognosis: A case report and review of the literature

**DOI:** 10.3892/ol.2013.1446

**Published:** 2013-07-05

**Authors:** JINGJIE ZHAO, XUEDONG ZHANG, JIANWEI LIU, JIANHUA LI

**Affiliations:** 1 Department of Pathology, Chengde Central Hospital, Chengde, Hebei 067000, P.R. China; 2 Department of Critical Care Medicine, Chengde Central Hospital, Chengde, Hebei 067000, P.R. China; 3 Department of Cardiothoracic Surgery, Chengde Central Hospital, Chengde, Hebei 067000, P.R. China; 4 Department of Oncology, Chengde Central Hospital, Chengde, Hebei 067000, P.R. China

**Keywords:** breast, osteosarcoma, chondroblastic, prognosis

## Abstract

The present study describes the case of a 77-year-old female with a recently self-detected, painless, 7-cm lump in the left breast, without evidence of metastasis clinically, who underwent mastectomy with dissection of the axillary lymph nodes. The tumor did not invade the chest wall and skin. The tumor was comprised of abundant chondroid matrix and fibrous tissue, with focal osteoid matrix, and was classified as a chondroblastic/fibroblastic variant. The tumor had a reverse zonal pattern. The tumor cells in the central portion were mainly spindle-like and sparse with minimal cytological atypia, while the remaining tumor cells in the periphery were mainly epithelioid, atypical and dense. Neoplastic osteoid woven bone or trabeculae were observed in the central portion of the tumor. No metastasis was identified in the axillary lymph nodes. The patient was alive without evidence of local recurrence or hematogenous spread at the 60-month follow-up.

## Introduction

Primary osteosarcoma of the breast is a rare tumor, which is indistinguishable from conventional osteosarcoma of the bone and other extraskeletal sites using histological examination (1–6). In comparison, bone-producing spindle cell neoplasms with an epithelial origin, known as metaplastic (sarcomatoid) carcinomas, and malignant phyllodes tumors are more common (7). Primary breast osteosarcomas are considered to be highly aggressive tumors that are associated with early recurrence and a tendency for hematogenous, instead of lymphatic, spread, most commonly to the lungs (2,3,6,8–11). The tumor subtype, size and mitotic figures may be predictors of prognosis. The present study describes a case of a chondroblastic/fibroblastic variant of this rare tumor that had a good prognosis.

## Case report

A 77-year-old Chinese woman presented to the Chengde Central Hospital Outpatient Department with complaints of a lump in the left breast that was self-detected a month prior to presentation. There was no history of nipple discharge, fever and pain. There was no history of breast trauma, prior local irradiation and surgery, nor any other tumor history. The patient denied using any hormonal therapy or a family history of breast disease. A breast examination showed a 7×7×6-cm irregular, firm mass in the lower inner quadrant of the left breast. The mass was poorly mobile and adherent to the skin and chest wall. No axillary lymphadenopathy was detected upon physical examination. Mammography showed that the mass was relatively well demarcated and partially calcified. The tumor did not invade the overlying skin and underlying chest wall. Breast carcinoma was thus indicated. The patient refused a needle biopsy and underwent a mastectomy.

The mastectomy specimen contained a 7-cm, relatively well-circumscribed mass, which had a gray-white cartilaginous to firm calcified appearance in the cross-section.

**Figure 1:**
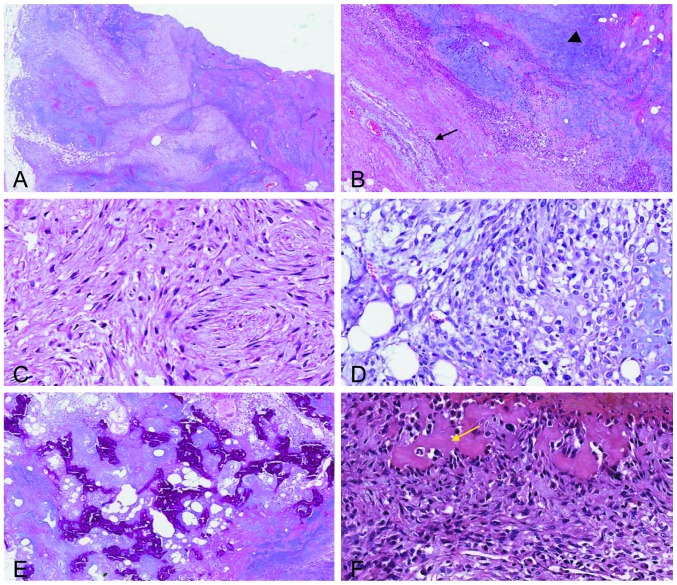
(A) The tumor was relatively well-demarcated, but the adjacent fat tissue had been invaded (magnification, ×10). (B) The surrounding non-neoplastic breast parenchyma revealed compressed lobular units (arrow) (magnification, ×40). The tumor was mainly composed of a cartilaginous component (arrowhead). (C) The tumor cells in the central portion were mainly spindle-like and sparse with minimal cytological atypia (magnification, ×200). (D) The tumor cells in the periphery were mainly epithelioid, atypical and dense (magnification, ×200). Low mitotic activity was identified even in the dense area. Neoplastic osteoid woven bone or trabeculae (arrow) may be observed at (E) ×40 magnification and (F) ×200 magnification.

Microscopically, the tumor was slightly lobular and relatively well demarcated, however, the adjacent fat tissue had been invaded and the surrounding non-neoplastic breast parenchyma revealed compressed lobular units (Fig. 1A and B). The tumor was mainly composed of cartilaginous components. The abundant cartilaginous proliferation varied from mature lacunar cartilage to poorly-differentiated areas displaying myxoid changes with no lacunar arrangement. In certain areas there was a transition from cartilaginous proliferation to fibrous cells. More than half of the tumor cells (~60%) were spindle-like and sparse with minimal cytological atypia, which were mainly observed in the central portion, while the other cells (40%), which were mainly in the periphery, were epithelioid, atypical and dense. High-power magnification revealed low mitotic activity even in the dense area (1 mitoses/10 high power microscopic fields; Fig. 1C and D). Neoplastic osteoid woven bone or trabeculae were observed in the central portion (Fig. 1E and F). Hemorrhage and necrosis foci were also observed in the central portion. No lymph-vascular invasion or neural invasion was observed. There was no histological evidence of an epithelial or carcinomatous component, despite extensive sampling of the tumor. No evidence of a preexisting malignant phyllodes tumor was present. The tumor was negative for cytokeratin, as well as for the estrogen and progesterone receptors and HER2. The tumor was classified as a chondroblastic/fibroblastic variant of osteosarcoma. The 15 axillary lymph nodes studied showed no metastasis. The patient underwent a simple mastectomy without post-operative adjuvant chemotherapy or radiation therapy. At 60 months post-mastectomy, the patient was alive and well without clinical evidence of local recurrence or distant metastasis. Written informed consent was obtained from the patient for publication of this case report and all accompanying images.

## Discussion

Carcinoma is the most common malignancy of the breast and sarcomas form a minority of breast neoplasms. Primary osteosarcoma of the breast is rare and represents <1% of all primary breast malignancies. However, the actual incidence of primary osteosarcoma is difficult to determine, as a number of the ~100 previously-reported cases are likely to have included metaplastic carcinomas, as well as osteogenic sarcomas arising in association with a biphasic tumor, such as a phyllodes tumor or carcinosarcoma (7). Almost every previous reference to primary osteosarcoma of the breast in the literature is in the form of single case reports. Silver and Tavassoli reported a clinicopathological analysis of 50 cases observed over a 38-year period, the largest collection of primary breast osteogenic sarcomas to date (6). In almost all cases, the patients were diagnosed clinically as having breast carcinoma and the final diagnosis was established by histology. The histogenesis of primary osteosarcoma of the breast remains unclear, but an origin from totipotent mesenchymal cells of the breast stroma or a transformation from a preexisting fibroadenoma or phyllodes tumor has been suggested (1).

The presentation of breast osteosarcoma usually occurs at an advanced age, in contrast with skeletal osteosarcomas where the patients are younger. There has been a report of a breast osteosarcoma diagnosis at the age of 96 years, however, the usual mean age at presentation has been reported to be ~64 years, and cases are more frequently postmenopausal (1,6). Risk factors for extraskeletal osteosarcomas have not been identified to date, although certain cases have been attributed to local irradiation, trauma or the presence of a foreign body (6). The majority of patients present with a mobile, often large, irregular lump, without axillary metastases. Primary osteosarcomas should be separated from malignant phyllodes tumors with malignant heterologous differentiation. Other diagnoses that should be excluded are: i) osteosarcomatous differentiation in carcinoma of the breast (metaplastic carcinoma), which are likely to be myoepithelial differentiation; ii) osteogenic sarcoma arising from the underlying ribs or sternum; and iii) metastatic osteosarcoma. The present patient was 77 years old with no history of trauma or irradiation, no tumor in other sites and absence of a biphasic image in the breast. Other diagnoses were therefore excluded, and it was concluded that the patient had primary osteosarcoma of the breast.

The reverse zonation pattern is observed in half of all breast osteosarcomas (the majority of fibroblastic types) (6). In the zonation pattern, the bone tissue at the peripheral region is more mature than that in the central region, which is generally the phenomenon observed in myositis ossificans. In the reverse zonation pattern, the central portion of the tumor was largely composed of neoplastic osteoid woven bone or trabeculae, with sarcomatous stroma confined to the periphery of the neoplasm, imparting a zonal pattern (the reverse of that observed in myositis ossificans). A reverse zonation pattern was observed in the present case. In addition, central necrosis has been noted in 10% of all breast osteosarcomas. Generally, central necrosis is observed in large tumors, such as tumors with surface ulceration (6). Areas of necrosis were identified in the center of the tumor in the present case.

Primary breast osteosarcomas are considered to be highly aggressive tumors associated with early recurrence and a tendency for hematogenous, instead of lymphatic, spread, most commonly to the lungs (2,3,6,8–11).

It has been reported that the predictive factors for outcome in this disease are tumor size, histological grade and subtype. Silver and Tavassoli (6) observed that mammary osteosarcomas that were on average 4.6 cm in diameter were associated with a significantly higher survival rate than larger tumors. Histological differentiation is important since fibroblastic osteosarcomas have a improved survival outcome compared with other pathological types, in breast osteosarcomas as well as in primary bone and soft tissue osteosarcomas. The histological appearance of primary osteosarcoma of the breast varies according to the cellular composition (fibroblastic, osteoblastic and osteoclastic), as well as the type and amount of the matrix (osteoid, osseous and chondroid). Abundant chondrosarcomatous components are rarely observed (6,12). Silver and Tavassoli (6) classified primary osteosarcoma of the breast into the fibroblastic, osteoblastic and osteoclastic types. The osteoclastic type is not in the current World Health Organization (WHO) classification of osteosarcomas (13). Silver and Tavassoli (6) report that this subtype contains abundant, multi-nucleated osteoclast giant cells diffusely scattered within the sarcomatous stroma or surrounding osteoid islands. The fibroblastic type constituted the majority of cases (56%) in their study, while the osteoblastic type was the smallest subtype (16%). Patients with osteosarcoma of the fibroblastic subtype have a significantly improved 5-year survival rate (67%) than those with osteosarcoma of the osteoclastic or osteoblastic subtype (31%). Mitotic activity in osteosarcoma of the breast ranges between 5 and >20 mitotic figures/10 high-power fields. The osteosarcoma of the breast that has a high mitotic rate is limited to the osteoblastic and osteoclastic variants. This may be the reason for the different survival rates among subtypes. The present tumor was classified into the chondroblastic/fibroblastic variant, and the mitotic activity of this tumor was low. The reasons for the lack of recurrence in the 5 years subsequent to the surgery may be related to the subtype, minimal cytological atypia, low mitotic rate and good local control due to adequate resection, even though the tumor size was 7 cm.

Treatment for localized disease should include complete surgical removal of the tumor with an adequate margin. A simple mastectomy may be indicated to ensure complete excision of large tumors with cryptically infiltrative margins. Axillary lymph node dissection is not indicated in the setting of clinically-negative nodes, as axillary node involvement is rare. Whereas, for metastatic disease, chemotherapy based on the classic drugs (doxorubicine, ifosfamide, cisplatinium and methotrexate) used for osteosarcoma is the main treatment. Distinguishing metaplastic carcinoma and carcinosarcoma from osteosarcoma of the breast is important, since the former requires treatment as a primary breast cancer.

The majority of reported primary breast osteosarcomas are considered highly aggressive tumors associated with early recurrence and a propensity for hematogenous spread. However, the presence of a chondroblastic/fibroblastic variant, minimal cytological atypia, a low mitotic rate and good local control due to adequate resection may result in a good prognosis.
